# Supplementary material for: Longitudinal Outcomes of Gender Identity in Children (LOGIC): protocol for a prospective longitudinal cohort study of children referred to the UK gender identity development service
Source: BMJ Open. 2021 Sep 7;11(9):e045628. doi: 10.1136/bmjopen-2020-045628 (PMC8424855; doi:10.1136/bmjopen-2020-045628)
Supplement: Author's manuscript [file bmjopen-2020-045628.draft_revisions.pdf]

BMJ Open

BMJ Open is committed to open peer review. As part of this commitment we make the peer review history of every article we publish publicly available.

When an article is published we post the peer reviewers' comments and the authors' responses online. We also post the versions of the paper that were used during peer review. These are the versions that the peer review comments apply to.

The versions of the paper that follow are the versions that were submitted during the peer review process. They are not the versions of record or the final published versions. They should not be cited or distributed as the published version of this manuscript.

BMJ Open is an open access journal and the full, final, typeset and author-corrected version of record of the manuscript is available on our site with no access controls, subscription charges or pay-per-view fees (http://bmjopen.bmj.com).

If you have any questions on BMJ Open's open peer review process please email info.bmjopen@bmj.com

BMJ Open

Longitudinal Outcomes of Gender Identity in Children (LOGIC): protocol for a prospective longitudinal cohort study of children referred to the UK gender identity development service

Journal:	*BMJ Open*
Manuscript ID	bmjopen-2020-045628
Article Type:	Protocol
Date Submitted by the Author:	06-Oct-2020
Complete List of Authors:	Kennedy, Eilis; Tavistock and Portman NHS Foundation Trust, Research & Development; University College London, Research Department of Clinical Educational and Health Psychology<br>Spinner, Lauren; Tavistock and Portman NHS Foundation Trust, Research & Development<br>Lane, Chloe; Tavistock and Portman NHS Foundation Trust, Research & Development<br>Stynes, Hannah; Tavistock and Portman NHS Foundation Trust, Research & Development<br>Ranieri, Veronica; Tavistock and Portman NHS Foundation Trust, Research & Development; University College London, Research Department of Clinical Educational and Health Psychology<br>Carmichael, Polly; Tavistock and Portman NHS Foundation Trust, Gender Identity Development Service<br>Omar, Rumana; University College London, Department of Statistical Science<br>Vickerstaff, Victoria; University College London, Research Department of Primary Care and Population Health; University College London, Division of Psychiatry, Faculty of Brain Sciences<br>Hunter, Rachael; University College London, Research Department of Primary Care and Population Health<br>Wright, Talen; London School of Hygiene and Tropical Medicine, Faculty of Public Health and Policy<br>Senior, Robert; Tavistock and Portman NHS Foundation Trust<br>Butler, Gary; University College London Hospitals NHS Foundation Trust, Department of Paediatric and Adolescent Endocrinology; Tavistock and Portman NHS Foundation Trust, Gender Identity Development Service<br>Baron-Cohen, Simon; University of Cambridge, Autism Research Centre, Department of Psychiatry<br>Young, Bridget; University of Liverpool, Institute of Population Health Sciences<br>King, Michael; University College London, Division of Psychiatry, Faculty of Brain Sciences
Keywords:	Child & adolescent psychiatry < PSYCHIATRY, Health economics < HEALTH SERVICES ADMINISTRATION & MANAGEMENT, Paediatric endocrinology < DIABETES & ENDOCRINOLOGY

SCHOLARONE™
Manuscripts

I, the Submitting Author has the right to grant and does grant on behalf of all authors of the Work (as defined in the below author licence), an exclusive licence and/or a non-exclusive licence for contributions from authors who are: i) UK Crown employees; ii) where BMJ has agreed a CC-BY licence shall apply, and/or iii) in accordance with the terms applicable for US Federal Government officers or employees acting as part of their official duties; on a worldwide, perpetual, irrevocable, royalty-free basis to BMJ Publishing Group Ltd ("BMJ") its licensees and where the relevant Journal is co-owned by BMJ to the co-owners of the Journal, to publish the Work in this journal and any other BMJ products and to exploit all rights, as set out in our licence.

The Submitting Author accepts and understands that any supply made under these terms is made by BMJ to the Submitting Author unless you are acting as an employee on behalf of your employer or a postgraduate student of an affiliated institution which is paying any applicable article publishing charge ("APC") for Open Access articles. Where the Submitting Author wishes to make the Work available on an Open Access basis (and intends to pay the relevant APC), the terms of reuse of such Open Access shall be governed by a Creative Commons licence – details of these licences and which Creative Commons licence will apply to this Work are set out in our licence referred to above.

Other than as permitted in any relevant BMJ Author's Self Archiving Policies, I confirm this Work has not been accepted for publication elsewhere, is not being considered for publication elsewhere and does not duplicate material already published. I confirm all authors consent to publication of this Work and authorise the granting of this licence.

For peer review only

LOGIC prospective longitudinal cohort protocol

Longitudinal Outcomes of Gender Identity in Children (LOGIC): protocol for a prospective longitudinal cohort study of children referred to the UK gender identity development service

Eilis Kennedy[1,2]; Lauren Spinner[1]; Chloe Lane[1]; Hannah Stynes[1]; Veronica Ranieri[1]; Polly Carmichael[3]; Rumana Z Omar[4]; Victoria Vickerstaff[5,10]; Rachael Hunter[4]; Talen Wright[6]; Rob Senior[1]; Gary Butler[3,7]; Simon Baron-Cohen[8]; Bridget Young[9]; Michael King[10]

[1] Research & Development Unit, Tavistock Centre, Tavistock and Portman NHS Foundation Trust

[2] Research Department of Clinical, Educational and Health Psychology, University College London

[3] Gender Identity Development Service, Tavistock and Portman NHS Foundation Trust

[4] Department of Statistical Science, University College London

[5] Research Department of Primary Care and Population Health, Royal Free Medical School, University College London

[6] Faculty of Public Health and Policy, London School of Hygiene and Tropical Medicine

[7] Department of Paediatric and Adolescent Endocrinology, University College London Hospital and University College London, Great Ormond Street Institute of Child Health

[8] Autism Research Centre, Department of Psychiatry, University of Cambridge

[9] Institute of Population Health Sciences, University of Liverpool

For peer review only

LOGIC prospective longitudinal cohort protocol

[10] Division of Psychiatry, Faculty of Brain Sciences, University College London

Address for correspondence: Dr Eilis Kennedy, Research & Development Unit, Tavistock Centre, Tavistock & Portman NHS Foundation Trust, 120 Belsize Lane, NW3 5AS, United Kingdom (e-mail: ekennedy@tavi-port.nhs.uk).

For peer review only

LOGIC prospective longitudinal cohort protocol

ABSTRACT

Introduction

Gender Identity Development Services (GIDS) worldwide have seen a significant increase in referrals in recent years. Many of these referrals consist of children and young people (CYP) who experience gender-related distress. This study aims to improve understanding of outcomes of CYP referred to the UK GIDS, specifically regarding gender identity, mental health, physical health and quality of life. Factors predicting outcomes, such as social transition and co-occurring autism, will be explored.

Methods and analysis

This is a prospective cohort study of CYP aged 3 to 14 years when referred to the UK GIDS. Eligible participants will be ≤14 years at the time their referral was accepted and will be on the waitlist for the service when baseline measures are completed. Children aged under 12 will complete the measures in an interview format with a researcher, whilst young people aged 12 and over, and their parents/caregivers will complete online or paper-based questionnaires. Participants will complete follow-up measures 12 months and 24 months later. The final sample size is expected to be approximately 500. Logistic regression models will be used to explore associations between pre-specified explanatory variables and gender dysphoria. Appropriate regression models will also be used to investigate explanatory variables for other clinical outcomes such as mental health, wellbeing, quality of life and medical treatment. Sub-group analyses based on birth-assigned gender, age at referral and co-occurring autistic traits will be explored.

Ethics and dissemination

The study has been approved by the UK Health Research Authority and London – Hampstead Research Ethics Committee (reference: 19/LO/0857). The study findings will be

For peer review only

published in peer-reviewed journals and presented at both conferences and stakeholder events. Findings will be used to inform clinical practice.

ARTICLE SUMMARY

Strengths and limitations

- This study will recruit a large cohort from one of the world's largest GIDS for children and young people.
- The prospective longitudinal cohort study design will enable documentation of the natural history and outcomes over time of children and young people referred to the UK GIDS.
- Data will be collected directly from both children and young people and their parent/caregiver.
- As this is a two-year prospective study, it will not be possible to ascertain longer-term health and wellbeing outcomes and treatment decisions.
- As the study is recruiting families on the GIDS waitlist, this is a potentially challenging population to access as they are not in receipt of clinical services at the time of recruitment.

For peer review only

LOGIC prospective longitudinal cohort protocol

INTRODUCTION

Gender identity development services for children and young people (CYP) have experienced a significant rise in referrals over recent years (1,2). In the UK gender identity development service (GIDS), this is evidenced by an increase from 1408 referrals of CYP in 2015/16 to 2728 referrals of CYP in 2019/20 (3). A changing pattern in referrals has also been observed and in particular, there has been a shift to an increase in referrals of CYP assigned female at birth (4,5). It has been noted that CYP referred to the GIDS are a heterogeneous group and whilst some report distress associated with a mismatch between their gender assigned at birth and their gender identity, others do not report such distress (6). Furthermore, some CYP who are referred to the service identify with a non-binary identity (6,7). Although these studies provide important insights into the characteristics of CYP who are referred to the GIDS, large scale studies assessing outcomes over time for these CYP are lacking and thus, longitudinal research is needed to further inform clinical practice.

Findings from a number of studies indicate that CYP referred to gender services often experience mental health difficulties, such as depression and anxiety (8–10). High rates of self-harm and suicidality are also common (10–12). Furthermore, co-occurring gender dysphoria and autism spectrum disorder has been reported for some CYP who are referred to gender services (13,14). As co-occurring conditions are frequently reported for gender diverse CYP, it is important to explore the extent to which these impact longer term outcomes and to consider how clinical services can best support the needs of these CYP.

An increasing number of younger children who have already socially transitioned (i.e. changed clothing, hairstyle, first name and pronouns to reflect their gender identity) at the time of referral to gender services has been reported (15). Recent research has suggested that this early social transition can have desirable outcomes for CYP, particularly in relation to a reduction in mental health difficulties (16,17). However, the longer term impacts of early social

For peer review only

transition in childhood have not yet been determined (18,19). Thus, prospective longitudinal research is needed to investigate outcomes in relation to early social transition, particularly in pre-pubertal children (20).

Current NHS intervention for CYP experiencing gender dysphoria is aimed at alleviating dysphoric feelings, improving wellbeing and supporting CYP and their families to make informed decisions about treatment and the meaning of gender identity within their own lives and contexts (6). However, the evidence on which current treatment protocols are based is widely acknowledged to be limited and predictors of individual gender and psychosexual developmental pathways are not clear (18). It can therefore be difficult to know which CYP referred to GIDS are more likely to access medical treatment (hormone blockers to suppress puberty or cross sex-hormones). In addition, factors that may predict the likelihood of receiving such treatment and the physical and psychological impacts of receiving this treatment have not yet been fully ascertained (21). Identifying these factors should provide much needed evidence to inform the care of gender diverse CYP attending services.

In summary, this study aims to improve understanding of the outcomes of CYP referred to the UK GIDS, specifically regarding gender identity, mental health, physical health and quality of life. The impact of factors such as co-occurring autism and early social transition on outcomes over time will be explored. The UK GIDS is one of the largest specialist gender services for CYP in the world and is therefore an ideal service from which to recruit. This is the first longitudinal study to assess outcomes of CYP referred to gender services in the UK and is novel in terms of recruiting families who are on the waitlist for the GIDS at baseline, the inclusion of a cohort of CYP who were ≤14 years at the time of referral and the commitment to follow up over time, regardless of whether families actually attend the service.

Aims

For peer review only

LOGIC prospective longitudinal cohort protocol

This study aims to investigate the outcomes of CYP referred to the UK GIDS and factors which predict these outcomes. Specifically, the study aims to establish 1) the proportion of CYP who experience ongoing gender dysphoria and access medical treatment; 2) factors which predict ongoing gender dysphoria and treatment choice; 3) how a) medical treatment, b) social transition and c) co-occurring autism, impact physical health, wellbeing, quality of life, peer and family relationships and cost to the NHS and other public services.

METHODS and ANALYSIS

Study design

A prospective longitudinal cohort study examining the outcomes of CYP referred to the UK GIDS. This study forms part of a wider programme of research investigating longitudinal outcomes of gender identity in children (the LOGIC study) (22). This programme of research utilises a mixed methods approach, incorporating both quantitative and qualitative longitudinal studies to investigate the experiences, outcomes and wellbeing of families referred to the UK GIDS. Please see LOGIC-Q protocol for further details on the longitudinal qualitative study.

Study Setting

The study is based at the Tavistock & Portman NHS Foundation Trust and is recruiting participants from the GIDS waitlist. The GIDS is a nationally commissioned service covering England, Wales, Northern Ireland and in part, Scotland and the Republic of Ireland, through a series of outreach clinics and main hubs in London and Leeds. The study start date was July 2019 and the anticipated end date is February 2023.

Participants and eligibility

Participants will consist of CYP aged 3 to 14 years at the time their referral to the GIDS was accepted, and their parent/caregiver. Inclusion criteria for CYP are: had a referral to the GIDS accepted; awaiting first appointment with the service when baseline measures are

For peer review only

completed; speak English; live in the UK. Families will still be eligible to participate in the study even if, after referral, they no longer wish to remain on the waitlist for an appointment with the GIDS. Exclusion criteria for CYP are: aged >14 years at the time their referral to the GIDS was accepted; already attended an appointment at the GIDS before baseline measures are completed; do not provide assent to take part; do not have parent/caregiver consent. Parents/caregivers of eligible CYP will be asked to provide informed consent in order to participate and must also be proficient in English and live within the UK.

Recruitment and consent

Potential participants will be identified from referrals received by the GIDS and recruitment will consist of a three-step process. Potential participants (CYP and their families) will receive a study invitation letter informing them of the study and inviting them to take part. At least three working days after the letter is sent, the family will be contacted by telephone by a member of the GIDS administrative team. The purpose of this telephone call is to highlight the study to potential participants and seek their consent to pass their contact details to the LOGIC research team. If potential participants agree for their contact details to be passed on, a researcher from the LOGIC research team will then contact them directly and provide them with information sheets, including an age-appropriate version for the CYP. Families will have the opportunity to discuss the study with the researcher and if they are happy to participate, arrangements will be made for completion of the baseline measures. Informed consent will be requested from all parents/caregivers at baseline, prior to completion of the assessment measures. All CYP will be asked to assent at baseline and follow-up time points. Consent will be requested and recorded on either a paper or online consent form, depending on how the measures are completed. Participants will be informed that they have the right to withdraw from the study at any time, without providing a reason.

Data collection and measures

For peer review only

LOGIC prospective longitudinal cohort protocol

Data will be collected from both the CYP and their parent/caregiver. However, in some circumstances (e.g. CYP does not wish to participate), data may only be collected from a parent/caregiver. Depending on the age of the CYP and participant preference, assessments will be carried out either in a face-to-face, video call or telephone interview format with a research assistant (this method will primarily be used with children <12 years and/or those with neurodevelopmental conditions), or via a self-report questionnaire in a paper or electronic format (via Qualtrics, an online survey platform). It is anticipated that the majority of participants aged ≥ 12 years and their parent/caregiver will complete the questionnaires online via the Qualtrics survey platform. In the event that a participant becomes distressed or discloses a risk/safeguarding concern during an assessment, the researchers will adhere to the study risk protocol.

Questionnaires and assessments will include measures of: gender dysphoria and gender identity; social transition; mental health and wellbeing; autistic traits; self-evaluation of puberty stage, height and weight; quality of life; peer and family relationships; socio-cultural and demographic factors; and health care and societal costs. Measures will be completed at baseline, and one- and two- year follow-up periods. For a detailed list of measures, please see Table 1.

[Insert Table 1 about here]

For participants who are referred to and attend an endocrinology clinic at University College London Hospitals or Leeds General Infirmary, access to routinely collected clinical measurements of body composition, bone density and age and blood test values will be requested. Participants will be given the option to consent to the research team accessing this

For peer review only

information and therefore, requests will only be made for participants who provide consent. As per current NHS treatment protocols, those receiving medical treatment with hormone blockers (gonadotropin-releasing hormone agonists (GnRHa)) and/or cross-sex hormones will be monitored for changes in blood count, renal and liver function, skeletal maturity and bone density and Tanner pubertal staging. These data will allow adult height predictions to be tracked and clinical measurements of body proportions, height, spinal height, shoulder and hip widths and body composition by bioelectrical impedance to be taken. For a list of these clinic derived variables, please see Table 2.

[Insert Table 2 about here]

Personal data provided by potential participants during the recruitment phase, prior to assessments, will be held in a password protected database on an encrypted NHS server at the Tavistock and Portman NHS Foundation Trust, accessible solely to the chief investigator and immediate research team. Pseudonyms, in the form of participant ID numbers, will be assigned to participants once they have agreed to complete the baseline measures. Participant ID numbers will be held in a password protected database on an encrypted NHS server at the Tavistock and Portman NHS Foundation Trust.

Sample size

An initial sample size target of 638 participants was calculated on the basis of recruiting 70% of eligible referrals of CYP aged ≤13 years to the GIDS over an 18 month period. Allowing for 20% attrition, the final sample size is expected to be 510 participants with complete follow-up data. Assuming the proportion of CYP with gender dysphoria to be 40%, a sample size of 510 will allow us to estimate 20 regression coefficients with adequate precision

For peer review only

using a logistic regression model, based on pre-specified explanatory variables (23). This sample size is also sufficient to investigate the association between these explanatory variables and other continuous clinical outcomes using a linear regression model (24).

During baseline recruitment, as our initial target of recruiting 70% of incoming referrals of CYP aged ≤13 years was not being achieved, our recruitment strategy was reviewed and the decision was made to recruit families on the GIDS waitlist and to increase the eligibility criteria to those aged ≤14 years when their referral to the GIDS was accepted. The waitlist will be divided into thirds based on referral date and recruitment will target each third equally in order to avoid possible biases of uptake and waiting time.

Analysis plan

Data will be analysed in STATA by the statistical team at UCL PRIMENT's Clinical Trials Unit. Pseudonymised datasets will be sent to the team via UCL's GDPR-compliant Data Safe Haven file transfer portal. Participant characteristics at baseline and outcomes will be presented using mean (SD), median (IQ range) or frequencies (proportion), as appropriate. Characteristics and outcomes of participants who drop out of the study will be described and compared to those with complete follow-up data in order to establish whether there are differences between these participants or factors which may have motivated them to leave the study.

Logistic regression models will be used to explore associations between the pre-specified explanatory variables and gender dysphoria. Appropriate regression models will also be used to investigate explanatory variables for other clinical outcomes (mental health, wellbeing, quality of life and medical treatment). Model assumptions regarding linear relationships and normality for continuous variables will be investigated. Any violation of these assumptions will be handled using appropriate statistical methods. Exploratory subgroup

For peer review only

analyses will be carried out for pre-pubertal children and CYP with autistic traits using descriptive statistics and appropriate regression models, where possible. Physical health, wellbeing and quality of life outcomes over time will be compared descriptively between those in receipt of hormone blockers (GnRHa) and those of a similar age/pubertal stage who progress through puberty without such intervention.

Resource use will be collected using the child and adolescent services use schedule (CA-SUS) which has been specifically adapted for this study. The CA-SUS collects information about service use in the past six months regarding heath care resource use, including: GIDS, private, primary and secondary care, mental health, school attendance, educational support, voluntary services and medication. Reponses to the CA-SUS will be costed using the Personal Social Services Resource Unit (PSSRU) (25), NHS reference costs (26), British National Formulary (27) and other published sources. Descriptive statistics for resource use and cost will be reported by CYP in active contact with the service, versus those who are not. Further subgroup analyses will explore resource use and cost by medical treatment, social transition, co-occurring autistic traits and pre-pubertal children. Key predictors of costs will be investigated by including pre-specified explanatory variables and 'active contact with GIDS' in appropriate general linear models (GLM). The most appropriate GLM will be chosen using the Akaike information criteria (AIC). This will allow additional cost or cost-savings associated with active GIDS contact to be estimated. Similar models will also be used to investigate whether active contact with GIDS is related to improvements in quality of life. This will be based on quality adjusted life years (QALYs) calculated using the CHU9D, the relevant tariff and the area under the curve.

Bias in relation to missing data will be explored. If required, logistic regression will be used to identify predictors of missingness and the analysis models will be adjusted for

For peer review only

predictors of missingness. Multiple imputation may be used to impute the missing values of the explanatory variables, if appropriate.

Data storage

All data will be held in a password protected database on an encrypted NHS server at the Tavistock and Portman NHS Foundation Trust. Data processing agreements and data sharing agreements are in place between the Tavistock & Portman NHS Foundation Trust and study collaborators. Data transferred to UCL for analysis will be retained in a GDPR-compliant Data Safe Haven which is security key and password protected.

Patient and Public Involvement

The LOGIC study was developed in collaboration with a service user co-applicant and co-author (TW), UK gender identity development service users and external stakeholders. CYP and their families using the UK GIDS were consulted regarding their priorities for research at GID Service User Family Days in London and Leeds. This informed the research proposal and application for funding which was shared and further reviewed with three parents of young people accessing the service. Throughout the study, the research team will host CYP and parent/caregiver advisory groups who will meet every six months to provide ongoing feedback and advice on the study. These will include families who are participating in the LOGIC study.

ETHICS and DISSEMINATION

This study has been approved by the Health Research Authority and London – Hampstead Research Ethics Committee (Reference: 19/LO/0857). The study findings will be published in peer-reviewed journals and presented at both conferences and stakeholder events. No identifiable data will feature in the dissemination of results. It is hoped that the findings will be used to inform clinical practice in the NHS and elsewhere.

For peer review only

LOGIC prospective longitudinal cohort protocol

REFERENCES

1. Chen M, Fuqua J, Eugster EA. Characteristics of Referrals for Gender Dysphoria Over a 13-Year Period. Journal of Adolescent Health. 2016 Mar 1;58(3):369–71.
2. Kaltiala R, Bergman H, Carmichael P, de Graaf NM, Egebjerg Rischel K, Frisén L, et al. Time trends in referrals to child and adolescent gender identity services: a study in four Nordic countries and in the UK. Nordic Journal of Psychiatry. 2020 Jan 2;74(1):40–4.
3. Referrals to GIDS, 2015-16 to 2019-20 [Internet]. [cited 2020 Jun 29]. Available from: https://gids.nhs.uk/number-referrals
4. Aitken M, Steensma TD, Blanchard R, VanderLaan DP, Wood H, Fuentes A, et al. Evidence for an Altered Sex Ratio in Clinic-Referred Adolescents with Gender Dysphoria. The Journal of Sexual Medicine. 2015;12(3):756–63.
5. de Graaf NM, Giovanardi G, Zitz C, Carmichael P. Sex Ratio in Children and Adolescents Referred to the Gender Identity Development Service in the UK (2009–2016). Arch Sex Behav. 2018 Jul 1;47(5):1301–4.
6. Butler G, De Graaf N, Wren B, Carmichael P. Assessment and support of children and adolescents with gender dysphoria. Arch Dis Child [Internet]. 2018 Apr 12 [cited 2020 Jun 29]; Available from: http://adc.bmj.com/lookup/doi/10.1136/archdischild-2018-314992
7. Twist J, de Graaf NM. Gender diversity and non-binary presentations in young people attending the United Kingdom's National Gender Identity Development Service. Clin Child Psychol Psychiatry. 2019 Apr 1;24(2):277–90.
8. Vries ALC de, Doreleijers TAH, Steensma TD, Cohen-Kettenis PT. Psychiatric comorbidity in gender dysphoric adolescents. Journal of Child Psychology and Psychiatry. 2011;52(11):1195–202.

For peer review only

9. Holt V, Skagerberg E, Dunsford M. Young people with features of gender dysphoria: Demographics and associated difficulties. Clin Child Psychol Psychiatry. 2016 Jan 1;21(1):108–18.
10. Spack NP, Edwards-Leeper L, Feldman HA, Leibowitz S, Mandel F, Diamond DA, et al. Children and Adolescents With Gender Identity Disorder Referred to a Pediatric Medical Center. Pediatrics. 2012 Mar 1;129(3):418–25.
11. Skagerberg E, Parkinson R, Carmichael P. Self-Harming Thoughts and Behaviors in a Group of Children and Adolescents with Gender Dysphoria. International Journal of Transgenderism. 2013 Apr 1;14(2):86–92.
12. Olson J, Schrager SM, Belzer M, Simons LK, Clark LF. Baseline Physiologic and Psychosocial Characteristics of Transgender Youth Seeking Care for Gender Dysphoria. Journal of Adolescent Health. 2015 Oct 1;57(4):374–80.
13. de Vries ALC, Noens ILJ, Cohen-Kettenis PT, van Berckelaer-Onnes IA, Doreleijers TA. Autism Spectrum Disorders in Gender Dysphoric Children and Adolescents. J Autism Dev Disord. 2010 Aug 1;40(8):930–6.
14. Skagerberg E, Di Ceglie D, Carmichael P. Brief Report: Autistic Features in Children and Adolescents with Gender Dysphoria. J Autism Dev Disord. 2015 Aug 1;45(8):2628–32.
15. Steensma TD, Cohen-Kettenis PT. Gender Transitioning before Puberty? Arch Sex Behav. 2011 Aug 1;40(4):649–50.
16. Durwood L, McLaughlin KA, Olson KR. Mental Health and Self-Worth in Socially Transitioned Transgender Youth. Journal of the American Academy of Child & Adolescent Psychiatry. 2017 Feb 1;56(2):116-123.e2.
17. Olson KR, Durwood L, DeMeules M, McLaughlin KA. Mental Health of Transgender Children Who Are Supported in Their Identities. Pediatrics [Internet]. 2016 Mar 1 [cited

For peer review only

2020 Jun 23];137(3). Available from: https://pediatrics.aappublications.org/content/137/3/e20153223

18. Olson-Kennedy J, Cohen-Kettenis PT, Kreukels BPC, Meyer-Bahlburg HFL, Garofalo R, Meyer W, et al. Research Priorities for Gender Nonconforming/Transgender Youth: Gender Identity Development and Biopsychosocial Outcomes. Curr Opin Endocrinol Diabetes Obes. 2016 Apr;23(2):172–9.
19. Turban JL. Transgender Youth: The Building Evidence Base for Early Social Transition. Journal of the American Academy of Child & Adolescent Psychiatry. 2017 Feb 1;56(2):101–2.
20. Olson KR. Prepubescent Transgender Children: What We Do and Do Not Know. Journal of the American Academy of Child & Adolescent Psychiatry. 2016 Mar 1;55(3):155-156.e3.
21. Butler G, Wren B, Carmichael P. Puberty blocking in gender dysphoria: suitable for all? Archives of Disease in Childhood. 2019 Jun 1;104(6):509–10.
22. Longitudinal Outcomes of Gender Identity in Children [Internet]. The LOGIC study. [cited 2020 Apr 22]. Available from: https://logicstudy.uk/
23. Peduzzi P, Concato J, Kemper E, Holford TR, Feinstein AR. A simulation study of the number of events per variable in logistic regression analysis. Journal of Clinical Epidemiology. 1996 Dec 1;49(12):1373–9.
24. Machin D, Campbell MJ, Tan SB, Tan SH. Sample Sizes for Clinical, Laboratory and Epidemiology Studies. John Wiley & Sons; 2018. 406 p.
25. Curtis LA, Burns A. Unit Costs of Health and Social Care 2019 [Internet]. Kent, UK: PSSRU; 2019 [cited 2020 Oct 5]. 176 p. Available from: https://doi.org/10.22024/UniKent/01.02.79286

For peer review only

LOGIC prospective longitudinal cohort protocol

26. Archived Reference Costs | NHS Improvement [Internet]. [cited 2020 Oct 5]. Available from: https://improvement.nhs.uk/resources/reference-costs/

27. Pharmaceutical Press - British National Formulary Online via MedicinesComplete [Internet]. [cited 2020 Oct 5]. Available from: https://www.pharmpress.com/product/MC_BNF/british-national-formulary

28. Martin CL, Andrews NCZ, England DE, Zosuls K, Ruble DN. A Dual Identity Approach for Conceptualizing and Measuring Children's Gender Identity. Child Development. 2017;88(1):167–82.

29. Cohen-kettenis PT, Van goozen SHM. Sex Reassignment of Adolescent Transsexuals: A Follow-up Study. Journal of the American Academy of Child & Adolescent Psychiatry. 1997 Feb 1;36(2):263–71.

30. Timmins L, Rimes KA, Rahman Q. Minority stressors and psychological distress in transgender individuals. Psychology of Sexual Orientation and Gender Diversity. 2017 Sep;4(3):328–40.

31. Achenbach, TM. The Achenbach system of empirically based assessemnt (ASEBA): development, findings, theory, and applications. Burlington, VT: University of Vermont Research Center for Children, Youth and Families; 2009.

32. Baron-Cohen S, Wheelwright S, Skinner R, Martin J, Clubley E. The Autism-Spectrum Quotient (AQ): Evidence from Asperger Syndrome/High-Functioning Autism, Malesand Females, Scientists and Mathematicians. J Autism Dev Disord. 2001 Feb 1;31(1):5–17.

33. Goodman R. Psychometric Properties of the Strengths and Difficulties Questionnaire. Journal of the American Academy of Child & Adolescent Psychiatry. 2001 Nov 1;40(11):1337–45.

For peer review only

34. Angold A, Costello EJ, Messer SC, Pickles A. Development of a short questionnaire for use in epidemiological studies of depression in children and adolescents. International Journal of Methods in Psychiatric Research. 1995;5(4):237–49.

35. Tennant R, Hiller L, Fishwick R, Platt S, Joseph S, Weich S, et al. The Warwick-Edinburgh Mental Well-being Scale (WEMWBS): development and UK validation. Health Qual Life Outcomes. 2007 Nov 27;5(1):63.

36. Liebenberg L, Ungar M, LeBlanc JC. The CYRM-12: A Brief Measure of Resilience. Can J Public Health. 2013 Mar 1;104(2):e131–5.

37. Byford S, Harrington R, Torgerson D, Kerfoot M, Dyer E, Harrington V, et al. Cost-effectiveness analysis of a home-based social work intervention for children and adolescents who have deliberately poisoned themselves: Results of a randomised controlled trial. Br J Psychiatry. 1999 Jan;174(1):56–62.

38. Ravens-Sieberer U, Gosch A, Rajmil L, Erhart M, Bruil J, Duer W, et al. KIDSCREEN-52 quality-of-life measure for children and adolescents. Expert Review of Pharmacoeconomics & Outcomes Research. 2005 Jun 1;5(3):353–64.

39. Stevens K. Assessing the performance of a new generic measure of health-related quality of life for children and refining it for use in health state valuation. Appl Health Econ Health Policy. 2011 May 1;9(3):157–69.

For peer review only

LOGIC prospective longitudinal cohort protocol

Author contributions: EK, PC, RO, RH, TW, RS, GB, SB-C, BY & MK contributed to the conception and design of the protocol. EK is the chief investigator of the LOGIC study. EK, LS, CL, HS & VR drafted the manuscript. RO & VV provided expertise on statistical analysis. RH provided expertise on health economic analysis. All authors drafted or critically revised the protocol and approved the final version of the manuscript.

Funding statement: This work was supported by the National Institute for Health Research, Health Services and Delivery Research grant number 17/51/19.

Competing interests: The authors declare no competing interests.

Word Count: 2812 (excluding abstract, tables and references)

For peer review only

LOGIC prospective longitudinal cohort protocol

Table 1. Summary of assessment measures to be completed at each time point

Measure	Informant /Source	Baseline	12 months	24 months
Demographics/Background	P	x		
**Gender Identity**				
Parent Questionnaire	P	x	x	x
Child Semi-Structured Interview	C	x	x	x
Young Person Questionnaire	YP	x	x	x
Gender Similarity Task (28)	C	x	x	x
Gender Diversity Questionnaire (GDQ) (7)	YP	x	x	x
Utrecht Gender Dysphoria Scale (UGDS) (29)	YP	x	x	x
Gender Identity Self Stigma Scale (30)	YP	x	x	x
**CYP Functioning**				
Child Behaviour Checklist (CBCL) (31)	P	x	x	x
Autism Quotient (AQ) (32)	P	x	x	x
Strengths and Difficulties Questionnaire (SDQ) (33)	P, YP	x	x	x
Youth Self Report (YSR) (31)	YP	x	x	x
Moods and Feelings Questionnaire (MFQ) (34)	YP	x	x	x
Warwick-Edinburgh Mental Wellbeing Scale (WEMWBS) (35)	YP	x	x	x
Child and Youth Resilience Measure (CYRM) (36)	C, YP	x	x	x
**Health Care Resource Use**				
Child & Adolescent Service Use Schedule (CA-SUS) (37)	P	x	x	x
**Quality of Life**				
Kidscreen-52 (38)	P or C, YP	x	x	x

For peer review only

Child Health Utility 9 Dimensions (CHU-9D) (39)	P or C, YP	x	x	x
**Physical Measures**				
Parental Height	P	x		
CYP Pubertal development	P, YP	x	x	x
CYP Height, Weight, BMI	P, YP	x	x	x

Child (C) ages for data collection are 3-11 years. Young people (YP) ages for data collection are ≥12 years. Parental (P) proxy measures are available for children (aged <8 years) not able to self-complete quality of life instruments.

For peer review only

LOGIC prospective longitudinal cohort protocol

Table 2 – Endocrinology clinic derived measurements for young people receiving medical treatment

	Data Source	Initial Assessment	FU 3-6m on GnRHa	FU 12m on GnRHa (monitoring cycle repeats)
**Auxology/Physical Measures**				
Height, Sitting Height, Bi-acromial width, Bi-iliac width, Weight	Clinical measures	x	x	x
BMI	Value derived by calculation	x	x	x
Parents height	Clinical measure	x		
Pubertal Tanner Staging	Clinical examination	x	x	x
Body Composition Assessment	Tanita bioelectrical impedance analyser	x	x	x
**Radiological Measures**				
Bone Densitometry	Dexa Scan	x	x	x
Bone Age	X-ray of left hand and wrist	x	x	x
**General Blood Measures**				
Full blood count, ferritin (iron)	Blood Test	x	x	x
Renal, Liver, Lipid and bone profiles	Blood Test	x	x	x
Vitamin D	Blood Test	x	x	x
**Endocrine Measures**				
Thyroid function test	Blood Test	x	x	x
Reproductive hormones: FSH, LH, PRL, Testosterone, Oestradiol	Blood Test	x	x	x

GnRHa – Gonadotropin-releasing hormone agonist; FSH – Follicle Stimulating Hormone, LH – Luteinising Hormone, PRL - Prolactin

For peer review only

BMJ Open

Longitudinal Outcomes of Gender Identity in Children (LOGIC): protocol for a prospective longitudinal cohort study of children referred to the UK gender identity development service

Journal:	*BMJ Open*
Manuscript ID	bmjopen-2020-045628.R1
Article Type:	Protocol
Date Submitted by the Author:	17-Feb-2021
Complete List of Authors:	Kennedy, Eilis; Tavistock and Portman NHS Foundation Trust, Research & Development; University College London, Research Department of Clinical Educational and Health Psychology<br>Spinner, Lauren; Tavistock and Portman NHS Foundation Trust, Research & Development<br>Lane, Chloe; Tavistock and Portman NHS Foundation Trust, Research & Development<br>Stynes, Hannah; Tavistock and Portman NHS Foundation Trust, Research & Development<br>Ranieri, Veronica; Tavistock and Portman NHS Foundation Trust, Research & Development; University College London, Research Department of Clinical Educational and Health Psychology<br>Carmichael, Polly; Tavistock and Portman NHS Foundation Trust, Gender Identity Development Service<br>Omar, Rumana; University College London, Department of Statistical Science<br>Vickerstaff, Victoria; University College London, Research Department of Primary Care and Population Health; University College London, Division of Psychiatry, Faculty of Brain Sciences<br>Hunter, Rachael; University College London, Research Department of Primary Care and Population Health<br>Wright, Talen; University College London, Division of Psychiatry<br>Senior, Robert; Tavistock and Portman NHS Foundation Trust<br>Butler, Gary; University College London Hospitals NHS Foundation Trust, Department of Paediatric and Adolescent Endocrinology; Tavistock and Portman NHS Foundation Trust, Gender Identity Development Service<br>Baron-Cohen, Simon; University of Cambridge, Autism Research Centre, Department of Psychiatry<br>Young, Bridget; University of Liverpool, Institute of Population Health Sciences<br>King, Michael; University College London, Division of Psychiatry, Faculty of Brain Sciences
**Primary Subject Heading**:	Mental health
Secondary Subject Heading:	Health services research

Keywords:	Child & adolescent psychiatry < PSYCHIATRY, Health economics < HEALTH SERVICES ADMINISTRATION & MANAGEMENT, Paediatric endocrinology < DIABETES & ENDOCRINOLOGY

SCHOLARONE™
Manuscripts

I, the Submitting Author has the right to grant and does grant on behalf of all authors of the Work (as defined in the below author licence), an exclusive licence and/or a non-exclusive licence for contributions from authors who are: i) UK Crown employees; ii) where BMJ has agreed a CC-BY licence shall apply, and/or iii) in accordance with the terms applicable for US Federal Government officers or employees acting as part of their official duties; on a worldwide, perpetual, irrevocable, royalty-free basis to BMJ Publishing Group Ltd ("BMJ") its licensees and where the relevant Journal is co-owned by BMJ to the co-owners of the Journal, to publish the Work in this journal and any other BMJ products and to exploit all rights, as set out in our licence.

The Submitting Author accepts and understands that any supply made under these terms is made by BMJ to the Submitting Author unless you are acting as an employee on behalf of your employer or a postgraduate student of an affiliated institution which is paying any applicable article publishing charge ("APC") for Open Access articles. Where the Submitting Author wishes to make the Work available on an Open Access basis (and intends to pay the relevant APC), the terms of reuse of such Open Access shall be governed by a Creative Commons licence – details of these licences and which Creative Commons licence will apply to this Work are set out in our licence referred to above.

Other than as permitted in any relevant BMJ Author's Self Archiving Policies, I confirm this Work has not been accepted for publication elsewhere, is not being considered for publication elsewhere and does not duplicate material already published. I confirm all authors consent to publication of this Work and authorise the granting of this licence.

For peer review only

Longitudinal Outcomes of Gender Identity in Children (LOGIC): protocol for a prospective longitudinal cohort study of children referred to the UK gender identity development service

Eilis Kennedy[1,2]; Lauren Spinner[1]; Chloe Lane[1]; Hannah Stynes[1]; Veronica Ranieri[1]; Polly Carmichael[3]; Rumana Z Omar[4]; Victoria Vickerstaff[5,6]; Rachael Hunter[4]; Talen Wright[6]; Rob Senior[1]; Gary Butler[3,7]; Simon Baron-Cohen[8]; Bridget Young[9]; Michael King[6]

[1] Research & Development Unit, Tavistock Centre, Tavistock and Portman NHS Foundation Trust

[2] Research Department of Clinical, Educational and Health Psychology, University College London

[3] Gender Identity Development Service, Tavistock and Portman NHS Foundation Trust

[4] Department of Statistical Science, University College London

[5] Research Department of Primary Care and Population Health, Royal Free Medical School, University College London

[6] Division of Psychiatry, Faculty of Brain Sciences, University College London

[7] Department of Paediatric and Adolescent Endocrinology, University College London Hospital and University College London, Great Ormond Street Institute of Child Health

[8] Autism Research Centre, Department of Psychiatry, University of Cambridge

[9] Institute of Population Health Sciences, University of Liverpool

For peer review only

Address for correspondence: Dr Eilis Kennedy, Research & Development Unit, Tavistock Centre, Tavistock & Portman NHS Foundation Trust, 120 Belsize Lane, NW3 5AS, United Kingdom (e-mail: ekennedy@tavi-port.nhs.uk).

For peer review only

ABSTRACT

Introduction

Gender Identity Development Services (GIDS) worldwide have seen a significant increase in referrals in recent years. Many of these referrals consist of children and young people (CYP) who experience gender-related distress. This study aims to improve understanding of outcomes of CYP referred to the UK GIDS, specifically regarding gender identity, mental health, physical health and quality of life. The impact of factors such as co-occurring autism and early social transition on outcomes over time will be explored.

Methods and analysis

This is a prospective cohort study of CYP aged 3 to 14 years when referred to the UK GIDS. Eligible participants will be ≤14 years at the time their referral was accepted and will be on the waitlist for the service when baseline measures are completed. Children aged under 12 will complete the measures in an interview format with a researcher, whilst young people aged 12 and over, and their parents/caregivers will complete online or paper-based questionnaires. Participants will complete follow-up measures 12 months and 24 months later. The final sample size is expected to be approximately 500. Logistic regression models will be used to explore associations between pre-specified explanatory variables and gender dysphoria. Appropriate regression models will also be used to investigate explanatory variables for other outcomes. Sub-group analyses based on birth-assigned gender, age at referral and co-occurring autistic traits will be explored.

Ethics and dissemination

The study has been approved by the UK Health Research Authority and London – Hampstead Research Ethics Committee (reference: 19/LO/0857). The study findings will be

For peer review only

published in peer-reviewed journals and presented at both conferences and stakeholder events. Findings will be used to inform clinical practice.

ARTICLE SUMMARY

Strengths and limitations

- This study will recruit a large cohort from one of the world's largest GIDS for children and young people.
- The prospective longitudinal cohort study design will enable documentation of the natural history and outcomes over time of children and young people referred to the UK GIDS.
- Data will be collected directly from both children and young people and their parent/caregiver.
- As this is a two-year prospective study, it will not be possible to ascertain longer-term health and wellbeing outcomes and treatment decisions.
- As the study is recruiting families on the GIDS waitlist, this is a potentially challenging population to access as they are not in receipt of clinical services at the time of recruitment.

For peer review only

INTRODUCTION

Gender identity development services for children and young people (CYP) have experienced a significant rise in referrals over recent years (1,2). In the UK gender identity development service (GIDS), this is evidenced by an increase from 1408 referrals of CYP in 2015/16 to 2728 referrals of CYP in 2019/20 (3). A changing pattern in referrals has also been observed and in particular, there has been a shift to an increase in referrals of CYP assigned female at birth (4,5). It has been noted that CYP referred to the GIDS are a heterogeneous group and whilst some report distress associated with a mismatch between their gender assigned at birth and their gender identity, others do not report such distress (6). Furthermore, some CYP who are referred to the service identify with a non-binary identity (6,7). Although these studies provide important insights into the characteristics of CYP who are referred to the GIDS, large scale studies assessing outcomes over time for these CYP are lacking and thus, longitudinal research is needed to further inform clinical practice.

Findings from a number of studies indicate that CYP referred to gender services often experience mental health difficulties, such as depression and anxiety, with prevalence tending to increase with age (8–10). High rates of self-harm and suicidality are also common (10–12). Furthermore, co-occurring gender dysphoria and autism spectrum disorder has been reported for some CYP who are referred to gender services (13,14). As co-occurring conditions are frequently reported for gender diverse CYP, it is important to explore the extent to which these impact longer term outcomes and to consider how clinical services can best support the needs of these CYP.

An increasing number of younger children who have already socially transitioned (i.e. changed clothing, hairstyle, first name and pronouns to reflect their gender identity) at the time of referral to gender services has been reported (15). Recent research has suggested that this early social transition can have desirable outcomes for CYP, particularly in relation to a

For peer review only - http://bmjopen.bmj.com/site/about/guidelines.xhtml

reduction in mental health difficulties (16,17). However, the longer term impacts of early social transition in childhood have not yet been determined (18,19). Thus, prospective longitudinal research is needed to investigate outcomes in relation to early social transition, particularly in pre-pubertal children (20).

Current NHS intervention for CYP experiencing gender dysphoria is aimed at alleviating dysphoric feelings, improving wellbeing and supporting CYP and their families to make informed decisions about treatment and the meaning of gender identity within their own lives and contexts (6). However, the evidence on which current treatment protocols are based is widely acknowledged to be limited and predictors of individual gender and psychosexual developmental pathways are not clear (18). It can therefore be difficult to know which CYP referred to GIDS are more likely to access medical treatment (hormone blockers to suppress puberty or cross sex-hormones). In addition, factors that may be associated with the likelihood of receiving such treatment and the physical and psychological impacts of receiving this treatment have not yet been fully ascertained (21). Identifying these factors should provide much needed evidence to inform the care of gender diverse CYP attending services.

There is also limited research on the direct health care costs and indirect wider societal costs for CYP referred to gender services and this information is important for policy makers and service providers to consider when planning services. In the context of a finite health budget, reporting this information alongside health and quality of life gains provides information on the potential value for money of gender services, and how they might best be configured for maximum benefit to the health care service and wider society.

In summary, this study aims to improve understanding of the outcomes of CYP referred to the UK GIDS, specifically regarding gender identity, mental health, physical health and quality of life. The impact of factors such as co-occurring autism and early social transition on outcomes over time will be explored. The UK GIDS is one of the largest specialist gender

For peer review only

services for CYP in the world and is therefore an ideal service from which to recruit. This is the first longitudinal study to assess outcomes of CYP referred to gender services in the UK and is novel in terms of recruiting families who are on the waitlist for the GIDS at baseline, the inclusion of a cohort of CYP who were ≤14 years at the time of referral and the commitment to follow up over time, regardless of whether families actually attend the service.

Aims

This study aims to investigate the outcomes of CYP referred to the UK GIDS and the factors which are associated with these outcomes. Specifically, the study aims to establish 1) the proportion of CYP who experience ongoing gender dysphoria and access medical treatment; 2) factors which are associated with ongoing gender dysphoria and treatment choice; 3) how a) medical treatment, b) social transition and c) co-occurring autism, impact physical health, wellbeing, quality of life, peer and family relationships and cost to the NHS and other public services.

METHODS and ANALYSIS

Study design

A prospective longitudinal cohort study examining the outcomes of CYP referred to the UK GIDS. This study forms part of a wider programme of research investigating longitudinal outcomes of gender identity in children (the LOGIC study) (22). This programme of research utilises a mixed methods approach, incorporating both quantitative and qualitative longitudinal studies to investigate the experiences, outcomes and wellbeing of families referred to the UK GIDS. Please see the LOGIC-Q protocol for details on the longitudinal qualitative study, which involves semi-structured interviews with a purposively sampled sub-set of up to 40 families who have completed the quantitative assessments (McKay et al., under review).

Study Setting

For peer review only

The study is based at the Tavistock & Portman NHS Foundation Trust and is recruiting participants from the GIDS waitlist. The GIDS is a nationally commissioned service covering England, Wales, Northern Ireland and in part, Scotland and the Republic of Ireland, through a series of outreach clinics and main hubs in London and Leeds. The study start date was July 2019 and the anticipated end date is May 2023.

Participants and eligibility

Participants will consist of CYP aged 3 to 14 years at the time their referral to the GIDS was accepted, and their parent/caregiver. Referrals can be made by Child and Adolescent Mental Health Services (CAMHS), or from other health, social care or educational professionals, including GPs. The service does not accept self-referrals from CYP or their families. In order for the referral to be accepted, the referral must indicate that the CYP is experiencing concerns or distress around their gender identity. Inclusion criteria for CYP are: had a referral to the GIDS accepted; awaiting first appointment with the service when baseline measures are completed; speak English; live in the UK or the Republic of Ireland. Families will still be eligible to participate in the study even if, after referral, they no longer wish to remain on the waitlist for an appointment with the GIDS. Exclusion criteria for CYP are: aged >14 years at the time their referral to the GIDS was accepted; already attended an appointment at the GIDS before baseline measures are completed; do not provide assent to take part; do not have parent/caregiver consent. Parents/caregivers of eligible CYP will be asked to provide informed consent in order to participate and must also be proficient in English and live within the UK or the Republic of Ireland.

Recruitment and consent

Potential participants will be identified from referrals received by the GIDS and recruitment will consist of a three-step process. Potential participants (CYP and their families)

For peer review only

will receive a study invitation letter informing them of the study and inviting them to take part. At least three working days after the letter is sent, the family will be contacted by telephone by a member of the GIDS administrative team. The purpose of this telephone call is to highlight the study to potential participants and seek their consent to pass their contact details to the LOGIC research team. If potential participants agree for their contact details to be passed on, a researcher from the LOGIC research team will then contact them directly and provide them with information sheets, including an age-appropriate version for the CYP. Families will have the opportunity to discuss the study with the researcher and if they are happy to participate, arrangements will be made for completion of the baseline measures.

Informed consent will be requested from all parents/caregivers at baseline, prior to completion of the assessment measures. All CYP will be asked to assent at baseline and follow-up time points. Consent will be requested and recorded on either a paper or online consent form, depending on how the measures are completed. Participants will be informed that their decision to participate in the study (or not) will have no impact on their access to care, their position on the referral waiting list, or their future care pathway. Any clinical care team that the participant may go on to have will not know that the individual is taking part in the study unless they choose to share that information with them. Participation in the study will not be routinely documented on the participant's medical record. The only exception to this is if risk is identified for the child or young person, in which case the research team will follow a risk protocol which may result in a referral to the GIDS enquiries line to initiate support for the family. This information would be documented on the individual's medical record, but referrals such as this will only be made with the parent/caregiver's consent. Participants will be informed that they have the right to withdraw from the study at any time, without providing a reason, and without any impact on their access to care or their care pathway.

Data collection and measures

For peer review only

Data will be collected from both the CYP and their parent/caregiver. However, in some circumstances (e.g. CYP does not wish to participate), data may only be collected from a parent/caregiver. Depending on the age of the CYP and participant preference, assessments will be carried out either in a face-to-face, video call or telephone interview format with a research assistant (this method will primarily be used with children <12 years and/or those with neurodevelopmental conditions), or via a self-report questionnaire in a paper or electronic format (via Qualtrics, an online survey platform). It is anticipated that the majority of participants aged ≥12 years and their parent/caregiver will complete the questionnaires online via the Qualtrics survey platform. In the event that a participant becomes distressed or discloses a risk/safeguarding concern during an assessment, the researchers will adhere to the study risk protocol.

Questionnaires and assessments will include measures of: gender dysphoria and gender identity; social transition; mental health and wellbeing; autistic traits; self-evaluation of puberty stage, height and weight; quality of life; peer and family relationships; socio-cultural and demographic factors; and health care and societal costs. Measures will be completed at baseline, and one- and two- year follow-up periods. For a detailed list of measures, please see Table 1.

[Insert Table 1 about here]

For participants who are referred to and attend an endocrinology clinic at University College London Hospitals or Leeds General Infirmary, access to routinely collected clinical measurements of body composition, bone density and age and blood test values will be requested. Participants will be given the option to consent to the research team accessing this

For peer review only

information and therefore, requests will only be made for participants who provide consent. Clinicians and/or Endocrinologists will not know who is taking part in the study or for which participants this information is requested. These data will be collected by a member of the LOGIC research team with assistance from a research nurse at each site. As per current NHS treatment protocols, those receiving medical treatment with hormone blockers (gonadotropin-releasing hormone agonists (GnRHa)) and/or cross-sex hormones will be monitored for changes in blood count, renal and liver function, skeletal maturity and bone density and Tanner pubertal staging. These data will allow adult height predictions to be tracked and clinical measurements of body proportions, height, spinal height, shoulder and hip widths and body composition by bioelectrical impedance to be taken. For a list of these clinic derived variables, please see Table 2.

[Insert Table 2 about here]

Personal data provided by potential participants during the recruitment phase, prior to assessments, will be held in a password protected database on an encrypted NHS server at the Tavistock and Portman NHS Foundation Trust, accessible solely to the chief investigator and immediate research team. Pseudonyms, in the form of participant ID numbers, will be assigned to participants once they have agreed to complete the baseline measures. Participant ID numbers will be held in a password protected database on an encrypted NHS server at the Tavistock and Portman NHS Foundation Trust.

Sample size

It is estimated that 638 participants will be recruited over an 18 month period assuming a recruitment rate of 70% of all eligible referrals of CYP aged ≤13 years to the GIDS. Allowing

For peer review only

for 20% attrition, the final sample size with complete follow-up data is expected to be 510 participants. Assuming the proportion of CYP with gender dysphoria to be 40%, a sample size of 510 (number of GD events = 204) will allow us to estimate 20 regression coefficients with adequate precision using a logistic regression model, based on pre-specified explanatory variables (23). This sample size is more than sufficient to investigate the association between these explanatory variables and other continuous clinical outcomes using a linear regression model (24). For the analysis of the binary gender dysphoria outcome, if we are unable to obtain complete follow-up data from 510 participants we will limit the number of explanatory variables that will be included in the multivariable regression model. This decision will be based on the clinical importance/relevance of the variables and this will be pre-specified in the statistical analysis plan.

During baseline recruitment, as our initial target of recruiting 70% of incoming referrals of CYP aged ≤13 years was not being achieved, our recruitment strategy was reviewed and the decision was made to also recruit families on the existing GIDS waitlist and to increase the eligibility criteria to those aged ≤14 years when their referral to the GIDS was accepted. The families of all CYP who meet the eligibility criteria will be invited to participate in the study.

Analysis plan

Data will be analysed in STATA by the statistical team at UCL PRIMENT's Clinical Trials Unit. Pseudonymised datasets will be sent to the team via UCL's GDPR-compliant Data Safe Haven file transfer portal. Participant characteristics at baseline and outcomes will be presented using mean (SD), median (IQ range) or frequencies (proportion), as appropriate. Characteristics and outcomes of participants who drop out of the study will be described and compared descriptively to those with complete follow-up data in order to establish whether there are differences between these participants or factors which may have motivated them to leave the study.

For peer review only

A logistic regression model will be used to explore associations between the pre-specified explanatory variables and the binary gender dysphoria outcome. Linear regression models will be used for the continuous clinical outcomes. Appropriate regression models will be used to investigate explanatory variables for other outcomes. Models that account for repeated measurement data will be used where appropriate, including time period as an additional covariate. Model assumptions regarding linear relationships and normality for continuous variables will be investigated. Any violation of these assumptions will be handled using appropriate statistical methods. Exploratory subgroup analyses will be carried out for pre-pubertal children and CYP with autistic traits using descriptive statistics and appropriate regression models, where possible. Physical health, wellbeing and quality of life outcomes over time will be compared descriptively between those in receipt of hormone blockers (GnRHa) and those of a similar age/pubertal stage who progress through puberty without such intervention. Physical health data routinely collected by the endocrine service (see Table 2) will be reported descriptively.

Resource use will be collected using the child and adolescent services use schedule (CA-SUS) which has been specifically adapted for this study. The CA-SUS collects information about service use in the past six months regarding heath care resource use, including: GIDS, private, primary and secondary care, mental health, school attendance, educational support, voluntary services and medication. Reponses to the CA-SUS will be costed using the Personal Social Services Resource Unit (PSSRU) (25), NHS reference costs (26), British National Formulary (27) and other published sources. Descriptive statistics for resource use and cost (numbers, percentages, means and standard deviations per CYP) will be reported by CYP in active contact with the service, versus those who are not. Further subgroup analyses will explore resource use and cost by medical treatment, social transition, co-occurring autistic traits and pre-pubertal children. Key predictors of costs will be investigated

For peer review only

by including pre-specified explanatory variables and 'active contact with GIDS' in appropriate general linear models (GLM). The most appropriate GLM will be chosen using the Akaike information criteria (AIC). This will allow additional cost or cost-savings associated with active GIDS contact to be estimated. Similar models will also be used to investigate whether active contact with GIDS is related to improvements in quality of life. This will be based on quality adjusted life years (QALYs) calculated using the CHU9D, the relevant tariff and the area under the curve.

Bias in relation to missing data will be investigated. If required, logistic regression will be used to identify predictors of missingness and these predictors will be included in the analysis models as part of secondary analyses. Multiple imputation may be used to impute the missing values of the explanatory variables, if appropriate.

Data storage

All data will be held in a password protected database on an encrypted NHS server at the Tavistock and Portman NHS Foundation Trust. Data processing agreements and data sharing agreements are in place between the Tavistock & Portman NHS Foundation Trust and study collaborators. Data transferred to UCL for analysis will be retained in a GDPR-compliant Data Safe Haven which is security key and password protected.

Patient and Public Involvement

The LOGIC study was developed in collaboration with a service user co-applicant and co-author (TW), UK gender identity development service users and external stakeholders. CYP and their families using the UK GIDS were consulted regarding their priorities for research at GID Service User Family Days in London and Leeds. This informed the research proposal and application for funding which was shared and further reviewed with three parents of young people accessing the service. Throughout the study, the research team will host CYP and

For peer review only

parent/caregiver advisory groups who will meet every six months to provide ongoing feedback and advice on the study. These will include families who are participating in the LOGIC study.

ETHICS and DISSEMINATION

This study has been approved by the Health Research Authority and London – Hampstead Research Ethics Committee (Reference: 19/LO/0857). The study findings will be published in peer-reviewed journals and presented at both conferences and stakeholder events. No identifiable data will feature in the dissemination of results. It is hoped that the findings will be used to inform clinical practice in the NHS and elsewhere.

REFERENCES

1. Chen M, Fuqua J, Eugster EA. Characteristics of Referrals for Gender Dysphoria Over a 13-Year Period. Journal of Adolescent Health. 2016 Mar 1;58(3):369–71.
2. Kaltiala R, Bergman H, Carmichael P, de Graaf NM, Egebjerg Rischel K, Frisén L, et al. Time trends in referrals to child and adolescent gender identity services: a study in four Nordic countries and in the UK. Nordic Journal of Psychiatry. 2020 Jan 2;74(1):40–4.
3. Referrals to GIDS, 2015-16 to 2019-20 [Internet]. [cited 2020 Jun 29]. Available from: https://gids.nhs.uk/number-referrals
4. Aitken M, Steensma TD, Blanchard R, VanderLaan DP, Wood H, Fuentes A, et al. Evidence for an Altered Sex Ratio in Clinic-Referred Adolescents with Gender Dysphoria. The Journal of Sexual Medicine. 2015;12(3):756–63.
5. de Graaf NM, Giovanardi G, Zitz C, Carmichael P. Sex Ratio in Children and Adolescents Referred to the Gender Identity Development Service in the UK (2009–2016). Arch Sex Behav. 2018 Jul 1;47(5):1301–4.
6. Butler G, De Graaf N, Wren B, Carmichael P. Assessment and support of children and adolescents with gender dysphoria. Arch Dis Child [Internet]. 2018 Apr 12 [cited 2020

For peer review only

Jun 29]; Available from: http://adc.bmj.com/lookup/doi/10.1136/archdischild-2018-314992

7. Twist J, de Graaf NM. Gender diversity and non-binary presentations in young people attending the United Kingdom's National Gender Identity Development Service. Clin Child Psychol Psychiatry. 2019 Apr 1;24(2):277–90.
8. Vries ALC de, Doreleijers TAH, Steensma TD, Cohen-Kettenis PT. Psychiatric comorbidity in gender dysphoric adolescents. Journal of Child Psychology and Psychiatry. 2011;52(11):1195–202.
9. Holt V, Skagerberg E, Dunsford M. Young people with features of gender dysphoria: Demographics and associated difficulties. Clin Child Psychol Psychiatry. 2016 Jan 1;21(1):108–18.
10. Spack NP, Edwards-Leeper L, Feldman HA, Leibowitz S, Mandel F, Diamond DA, et al. Children and Adolescents With Gender Identity Disorder Referred to a Pediatric Medical Center. Pediatrics. 2012 Mar 1;129(3):418–25.
11. Skagerberg E, Parkinson R, Carmichael P. Self-Harming Thoughts and Behaviors in a Group of Children and Adolescents with Gender Dysphoria. International Journal of Transgenderism. 2013 Apr 1;14(2):86–92.
12. Olson J, Schrager SM, Belzer M, Simons LK, Clark LF. Baseline Physiologic and Psychosocial Characteristics of Transgender Youth Seeking Care for Gender Dysphoria. Journal of Adolescent Health. 2015 Oct 1;57(4):374–80.
13. de Vries ALC, Noens ILJ, Cohen-Kettenis PT, van Berckelaer-Onnes IA, Doreleijers TA. Autism Spectrum Disorders in Gender Dysphoric Children and Adolescents. J Autism Dev Disord. 2010 Aug 1;40(8):930–6.
14. Skagerberg E, Di Ceglie D, Carmichael P. Brief Report: Autistic Features in Children and Adolescents with Gender Dysphoria. J Autism Dev Disord. 2015 Aug 1;45(8):2628–32.

For peer review only

15. Steensma TD, Cohen-Kettenis PT. Gender Transitioning before Puberty? Arch Sex Behav. 2011 Aug 1;40(4):649–50.

16. Durwood L, McLaughlin KA, Olson KR. Mental Health and Self-Worth in Socially Transitioned Transgender Youth. Journal of the American Academy of Child & Adolescent Psychiatry. 2017 Feb 1;56(2):116-123.e2.

17. Olson KR, Durwood L, DeMeules M, McLaughlin KA. Mental Health of Transgender Children Who Are Supported in Their Identities. Pediatrics [Internet]. 2016 Mar 1 [cited 2020 Jun 23];137(3). Available from: https://pediatrics.aappublications.org/content/137/3/e20153223

18. Olson-Kennedy J, Cohen-Kettenis PT, Kreukels BPC, Meyer-Bahlburg HFL, Garofalo R, Meyer W, et al. Research Priorities for Gender Nonconforming/Transgender Youth: Gender Identity Development and Biopsychosocial Outcomes. Curr Opin Endocrinol Diabetes Obes. 2016 Apr;23(2):172–9.

19. Turban JL. Transgender Youth: The Building Evidence Base for Early Social Transition. Journal of the American Academy of Child & Adolescent Psychiatry. 2017 Feb 1;56(2):101–2.

20. Olson KR. Prepubescent Transgender Children: What We Do and Do Not Know. Journal of the American Academy of Child & Adolescent Psychiatry. 2016 Mar 1;55(3):155-156.e3.

21. Butler G, Wren B, Carmichael P. Puberty blocking in gender dysphoria: suitable for all? Archives of Disease in Childhood. 2019 Jun 1;104(6):509–10.

22. Longitudinal Outcomes of Gender Identity in Children [Internet]. The LOGIC study. [cited 2020 Apr 22]. Available from: https://logicstudy.uk/

For peer review only

23. Peduzzi P, Concato J, Kemper E, Holford TR, Feinstein AR. A simulation study of the number of events per variable in logistic regression analysis. Journal of Clinical Epidemiology. 1996 Dec 1;49(12):1373–9.

24. Machin D, Campbell MJ, Tan SB, Tan SH. Sample Sizes for Clinical, Laboratory and Epidemiology Studies. John Wiley & Sons; 2018. 406 p.

25. Curtis LA, Burns A. Unit Costs of Health and Social Care 2019 [Internet]. Kent, UK: PSSRU; 2019 [cited 2020 Oct 5]. 176 p. Available from: https://doi.org/10.22024/UniKent/01.02.79286

26. Archived Reference Costs | NHS Improvement [Internet]. [cited 2020 Oct 5]. Available from: https://improvement.nhs.uk/resources/reference-costs/

27. Pharmaceutical Press - British National Formulary Online via MedicinesComplete [Internet]. [cited 2020 Oct 5]. Available from: https://www.pharmpress.com/product/MC_BNF/british-national-formulary

28. Martin CL, Andrews NCZ, England DE, Zosuls K, Ruble DN. A Dual Identity Approach for Conceptualizing and Measuring Children's Gender Identity. Child Development. 2017;88(1):167–82.

29. Cohen-kettenis PT, Van goozen SHM. Sex Reassignment of Adolescent Transsexuals: A Follow-up Study. Journal of the American Academy of Child & Adolescent Psychiatry. 1997 Feb 1;36(2):263–71.

30. Timmins L, Rimes KA, Rahman Q. Minority stressors and psychological distress in transgender individuals. Psychology of Sexual Orientation and Gender Diversity. 2017 Sep;4(3):328–40.

31. Achenbach, TM. The Achenbach system of empirically based assessemnt (ASEBA): development, findings, theory, and applications. Burlington, VT: University of Vermont Research Center for Children, Youth and Families; 2009.

For peer review only

32. Baron-Cohen S, Wheelwright S, Skinner R, Martin J, Clubley E. The Autism-Spectrum Quotient (AQ): Evidence from Asperger Syndrome/High-Functioning Autism, Malesand Females, Scientists and Mathematicians. J Autism Dev Disord. 2001 Feb 1;31(1):5–17.

33. Goodman R. Psychometric Properties of the Strengths and Difficulties Questionnaire. Journal of the American Academy of Child & Adolescent Psychiatry. 2001 Nov 1;40(11):1337–45.

34. Angold A, Costello EJ, Messer SC, Pickles A. Development of a short questionnaire for use in epidemiological studies of depression in children and adolescents. International Journal of Methods in Psychiatric Research. 1995;5(4):237–49.

35. Tennant R, Hiller L, Fishwick R, Platt S, Joseph S, Weich S, et al. The Warwick-Edinburgh Mental Well-being Scale (WEMWBS): development and UK validation. Health Qual Life Outcomes. 2007 Nov 27;5(1):63.

36. Liebenberg L, Ungar M, LeBlanc JC. The CYRM-12: A Brief Measure of Resilience. Can J Public Health. 2013 Mar 1;104(2):e131–5.

37. Byford S, Harrington R, Torgerson D, Kerfoot M, Dyer E, Harrington V, et al. Cost-effectiveness analysis of a home-based social work intervention for children and adolescents who have deliberately poisoned themselves: Results of a randomised controlled trial. Br J Psychiatry. 1999 Jan;174(1):56–62.

38. Ravens-Sieberer U, Gosch A, Rajmil L, Erhart M, Bruil J, Duer W, et al. KIDSCREEN-52 quality-of-life measure for children and adolescents. Expert Review of Pharmacoeconomics & Outcomes Research. 2005 Jun 1;5(3):353–64.

39. Stevens K. Assessing the performance of a new generic measure of health-related quality of life for children and refining it for use in health state valuation. Appl Health Econ Health Policy. 2011 May 1;9(3):157–69.

For peer review only

Author contributions: EK, PC, RO, RH, TW, RS, GB, SB-C, BY & MK contributed to the conception and design of the protocol. EK is the chief investigator of the LOGIC study. EK, LS, CL, HS & VR drafted the manuscript. RO & VV provided expertise on statistical analysis. RH provided expertise on health economic analysis. All authors drafted or critically revised the protocol and approved the final version of the manuscript.

Funding statement: This work was supported by the National Institute for Health Research (NIHR), Health Services and Delivery Research grant number 17/51/19. The views expressed are those of the authors and not necessarily those of the NIHR or the Department of Health and Social Care.

Competing interests: The authors declare no competing interests.

Word Count: 3319 (excluding abstract, tables and references

For peer review only

Table 1. Summary of assessment measures to be completed at each time point

Measure	Data collected	Informant/ Source	Baseline	12 months	24 months
**Demographics/Background**	Caregiver: age, gender, education, occupation, sexual orientation, ethnicity, relationship to child<br>CYP: age, sex assigned at birth, ethnicity, age at referral, background to referral	P	x	x	x
**Gender Identity**					
Parent Questionnaire	DSM-5 criteria for Gender Dysphoria in child; Family experiences of child's gender identity; Gender expression and social transition; Body image and body distress	P	x	x	x
Child Semi-Structured Interview	Verbal identification with experienced gender; Gender expression and social transition; Age first realized and expressed gender identity; Body image and distress	C	x	x	x
Young Person Questionnaire	DSM-5 criteria for Gender Dysphoria; Distress around gender identity; Gender expression and social transition; Body image and body distress; Sexual orientation	YP	x	x	x

For peer review only

Gender Similarity Task (28)	Perceived similarity to own-gender and other-gender peers	C	x	x	x
Gender Diversity Questionnaire (GDQ) (7)	Verbal identification with expressed gender; Gender fluidity; Gender expression; Age first realized and expressed gender identity	YP	x	x	x
Utrecht Gender Dysphoria Scale (UGDS) (29)	Severity of gender dysphoria	YP	x	x	x
Gender Identity Self Stigma Scale (30)	Internalised transphobia and stigma	YP	x	x	x
**CYP Functioning**					
Child Behaviour Checklist (CBCL) (31)	Emotional reactivity; Anxious/Depressed; Somatic Complaints; Withdrawn; Sleep Problems; Attention Problems; Aggressive Behaviour.	P	x	x	x
Autism Quotient (AQ) (32)	Autistic traits in child or adolescent	P	x	x	x
Strengths and Difficulties Questionnaire (SDQ) (33)	Emotional symptoms; Conduct problems; Hyperactivity/inattention; Peer relationship problems; Prosocial behaviour	P, YP	x	x	x
Youth Self Report (YSR) (31)	Anxious-Depressed; Withdrawn/Depressed; Somatic Complaints; Social Problems; Thought	YP	x	x	x

For peer review only

	Problems; Attention Problems; Rule-Breaking Behavior; Aggressive Behavior				
Moods and Feelings Questionnaire (MFQ) (34)	Depressive symptoms in child or adolescent	YP	x	x	x
Warwick-Edinburgh Mental Wellbeing Scale (WEMWBS) (35)	Mental wellbeing	YP	x	x	x
Child and Youth Resilience Measure (CYRM) (36)	Brief screener for resilience processes in the lives of adolescents	C, YP	x	x	x
**Health Care Resource Use**					
Child & Adolescent Service Use Schedule (CA-SUS) (37)	All-cause uses of hospital, community-based, and private health and social care services	P	x	x	x
**Quality of Life**					
Kidscreen-52 (38)	Physical Well-being, Psychological Well-being, Moods and Emotions, Self-Perception, Autonomy, Parent Relations and Home Life, Social Support and Peers, School Environment, Social Acceptance (Bullying), and Financial Resources	P or C, YP	x	x	x
Child Health Utility 9 Dimensions (CHU-9D) (39)	Paediatric health related quality of life for use in economic evaluation (quality adjusted life years)	P or C, YP	x	x	x

For peer review only

**Physical Measures**					
Parental Height	Height in centimetres	P	x		
CYP Pubertal development	Tanner stage	P, YP	x	x	x
CYP Height, Weight, BMI	Height in centimetres; Weight in kilograms	P, YP	x	x	x

Child (C) ages for data collection are 3-11 years. Young people (YP) ages for data collection are ≥12 years. Parental (P) proxy measures are available for children (aged <8 years) not able to self-complete quality of life instruments.

For peer review only

Table 2 – Endocrinology clinic derived measurements for young people receiving medical treatment

	Data Source	Initial Assessment	FU 3-6m on GnRHa	FU 12m on GnRHa (monitoring cycle repeats)
**Auxology/Physical Measures**				
Height, Sitting Height, Bi-acromial width, Bi-iliac width, Weight	Clinical measures	x	x	x
BMI	Value derived by calculation	x	x	x
Parent's height	Clinical measure	x		
Pubertal Tanner Staging	Clinical examination	x	x	x
Body Composition Assessment	Tanita bioelectrical impedance analyser	x	x	x
**Radiological Measures**				
Bone Densitometry	Dexa Scan	x	x	x
Bone Age	X-ray of left hand and wrist	x	x	x
**General Blood Measures**				
Full blood count, ferritin (iron)	Blood Test	x	x	x
Renal, Liver, Lipid and bone profiles	Blood Test	x	x	x
Vitamin D	Blood Test	x	x	x
**Endocrine Measures**				
Thyroid function test	Blood Test	x	x	x
Reproductive hormones: FSH, LH, PRL, Testosterone, Oestradiol	Blood Test	x	x	x

GnRHa – Gonadotropin-releasing hormone agonist; FSH – Follicle Stimulating Hormone, LH – Luteinising Hormone, PRL - Prolactin

For peer review only
